# Profiling the metabolome of adenomyosis-associated infertility patients to predict the pregnancy outcome of frozen embryo transfer

**DOI:** 10.3389/fendo.2025.1625638

**Published:** 2025-08-25

**Authors:** Wen Zhang, Yidong Chen, Bing Han, Rong Li, Caihong Ma, Jie Qiao

**Affiliations:** ^1^ State Key Laboratory of Female Fertility Promotion, Center for Reproductive Medicine, Department of Obstetrics and Gynecology, Peking University, Third Hospital, Beijing, China; ^2^ National Clinical Research Center for Obstetrics and Gynecology, Peking University Third Hospital, Beijing, China; ^3^ Key Laboratory of Assisted Reproduction, Ministry of Education, Peking University, Beijing, China; ^4^ Beijing Key Laboratory of Reproductive Endocrinology and Assisted Reproductive Technology, Beijing, China; ^5^ Research Units of Comprehensive Diagnosis and Treatment of Oocyte Maturation Arrest, Chinese Academy of Medical Sciences, Beijing, China; ^6^ Peking-Tsinghua Center for Life Sciences, Academy for Advanced Interdisciplinary Studies, Peking University, Beijing, China

**Keywords:** adenomyosis, infertility, metabolomics, frozen embryo transfer, prediction model

## Abstract

**Objective:**

This study explores the metabolic profiles in the peripheral blood of infertile patients with adenomyosis (ADM) to identify key metabolites affecting pregnancy outcomes in these patients undergoing frozen embryo transfer (FET). Our goal is to create a metabolite-based clinical prediction model for pregnancy outcomes in adenomyosis-associated infertility.

**Methods:**

This prospective cohort study from the Reproductive Center at Peking University Third Hospital enrolled 94 infertile patients with adenomyosis and control (CTRL) patients undergoing FET. We divided these patients into four groups based on clinical pregnancy success: ADM-Success, ADM-Fail, CTRL-Success, and CTRL-Fail. We collected peripheral blood on the day of embryo transfer and analyzed metabolites using ultrahigh-performance liquid chromatography-tandem mass spectrometry. We compared metabolome differences among the four groups using bioinformatics and evaluated the diagnostic performance of metabolites for predicting pregnancy outcomes using receiver operating characteristic curves.

**Results:**

We found the metabolic differences between ADM-Success group and ADM-Fail group, and established a “5 metabolites + age” panel (5 metabolites combined with woman’s age panel), which could effectively predict pregnancy outcomes of adenomyosis patients, and the area under the curve was 0.879 (P<0.001). The 5 metabolites included Androsterone, Propionic acid, Glycocholic acid, 2,6-Dihydroxypurine, Deoxycorticosterone. And this study explored the metabolic differences between adenomyosis group and control group.

**Conclusions:**

A “5 metabolites + age” panel could effectively predict pregnancy outcomes of adenomyosis patients who undergoing FET. There were notable differences in plasma metabolic profiles between adenomyosis-associated infertility and control patients.

## Introduction

Adenomyosis is a uterine disorder characterized by ectopic endometrial-like tissue (stroma, glands, and fibroblasts) pathologically demonstrated in the myometrium, causing hyperplasia and hypertrophy in the surrounding smooth muscle cells and local inflammatory responses. Adenomyosis patients may present with heavy menstrual bleeding, dysmenorrhea, dyspareunia, chronic pelvic pain, and infertility (1, 2).

Adenomyosis lesions disrupt the embryo implantation environment and endometrial decidualization, leading to alterations in endometrial receptivity genes, sex steroid hormone receptors, inflammatory molecules, extracellular matrix enzymes, growth factors, and myometrial contractility, thereby disrupting the process of embryo implantation (1, 3). Of these, there is not much evidence on the relevance of adenomyosis and metabolomics. Wei Song and other researchers have explored the metabolomic profiles of myometrium specimens from adenomyosis patients and control specimens using untargeted approach by combination of gas chromatography-mass spectrometry and high performance liquid chromatography-mass spectrometry, and finally found 106 metabolites differentially expressed in myometrium of adenomyosis, which mainly including nucleosides, lipids, amino acids, organic acids and carbohydrates. The above differential metabolites suggested that inflammation, oxidative stress, cell proliferation and apoptosis, and energy metabolism appeared to be involved in the progress of adenomyosis (4). Peipei Chen et al. obtained the fecal samples from adenomyosis-female ICR mice and control groups for microbial (16S rRNA gene sequencing) and metabolomic (liquid chromatography mass spectrometry, LC-MS) analysis. They found sixty differential expressed metabolites in intestinal metabolites, which were mainly involved in steroid hormone biosynthesis, cysteine and methionine metabolism, and alanine, aspartate, and glutamate metabolism (5).

However, few studies have explored the metabolic profile of peripheral blood around adenomyosis-associated infertile patients, as well as the correlation between metabolites and pregnancy outcomes of adenomyosis is not clear. Therefore, in this study, we enrolled patients with adenomyosis-associated infertility and control patients. We collected their peripheral blood on the day of embryo transfer and analyzed the peripheral blood metabolites using ultrahigh-performance liquid chromatography-tandem mass spectrometry. Thus, we aimed to investigate the metabolic profiles of peripheral blood in patients with adenomyosis-associated infertility, with the goal of identifying key metabolites that may influence pregnancy outcomes in these patients undergoing frozen embryo transfer (FET). What we are more interested in is to establish a metabolite-based clinical prediction model for pregnancy outcome in adenomyosis-associated infertile patients.

## Materials and methods

### Patients and cohorts

In order to characterize the metabolomic patterns of peripheral blood plasma from adenomyosis patients, we enrolled 94 infertile patients into this study who undergoing FET at the Reproductive Center of Peking University Third Hospital from August 2021 to June 2023, consisting of 51 adenomyosis patients and 43 control group patients. Inclusion criteria were as follow: (i) Adenomyosis was diagnosed by transvaginal ultrasound scans by two experienced senior radiologists. The control group were diagnosed with tubal obstruction-associated infertility by hysterosalpingography. (ii) Age range was between 18 and 42. (iii) Regular menstrual cycle. (iv) Cooperated with postoperative follow-up. Exclusion criteria were: (i) Patients with other serious endocrine diseases, immune diseases and tumors. (ii) Complicated with other factors influencing pregnancy, such as metabolic diseases, autoimmune diseases, chronic inflammatory diseases, uterine malformation and abnormal uterine cavity, decreased ovarian reserve, abnormal chromosomes in either husband or wife and so on. All participants provided their consent after being fully informed, and the Ethics Committee of Peking University Third Hospital granted approval (approval number: M20205007, approval date: 24/05/2021). Demographic and clinical information of all patients including age, primary infertility/secondary infertility, infertility duration, body mass index, Anti-Mullerian Hormone (AMH), antral follicle count (AFC), FET protocol, endometrial thickness, embryo/blastocyst, number of embryo/blastocyst, pregnancy outcome was recorded.

### *In vitro* fertilization-embryo transfer process

Different controlled ovarian hyperstimulation protocol were administrated for patients, such as GnRH-a ultralong protocol, GnRH-a long protocol, GnRH-antagonist protocol and so on (6). Either recombinant follicle-stimulating hormone (rFSH) or human menopausal gonadotrophins (hMG) were used. Standard methods for oocyte retrieval and fertilization with conventional *in vitro* fertilization (IVF) were used. Quality of embryos was evaluated according to the Istanbul Consensus Workshop on Embryo Assessment criteria (7). Embryos/blastocysts were transferred on Day 3/Day 5 in a fresh cycle, and the remained embryos/blastocysts were cryopreserved for FET. Blastocyst vitrification and thawing procedure were reported previously (8). For adenomyosis patients, GnRH-a were injected subcutaneously for 1–6 months or more before FET, and endometrial preparation was started 28 days after the last GnRH-a injection with estradiol and progesterone. A natural cycle/artificial cycle were applied for control patients. Peripheral blood samples from all patients were collected on the day of embryo transfer in their frozen embryo transfer (FET) cycles.

### Preprocessing of peripheral blood

Peripheral blood was collected from all subjects on the day of embryo or blastocyst transfer. Plasma was then isolated from the blood samples. Next, 100 μL of plasma was transferred into EP tubes and resuspended in prechilled 80% methanol using vigorous vortexing. The samples were incubated on ice for 5 minutes and subsequently centrifuged at 15,000 g and 4°C for 20 minutes. 80 μL of supernatant was diluted to a final concentration of 53% methanol using LC-MS grade water. The samples were then transferred to fresh Eppendorf tubes and centrifuged again at 15,000 g and 4°C for 20 minutes. Finally, the supernatant was injected into the LC-MS/MS system for analysis (9, 10).

### Untargeted metabolomics

Peripheral blood samples of patients were subjected to untargeted metabolomics conducted by a Thermo Syncronis C18 column (2.1 mm × 100 mm, 1.7 µm) coupled with an Orbitrap Q Exactive™ series mass spectrometer (Thermo Fisher Scientific). Samples were injected into a Hypesil Gold column (2.1 mm × 100 mm, 1.7 µm) and eluted with a 18-minute linear gradient at a flow rate of 0.2 mL/min. The mobile phase comprised eluents A (0.1% formic acid in water) and B (acetonitrile). The gradient profile was set as follows: 0–1 min, 95% A; 1–5 min, linear transition from 95% to 40% A; 5–8 min, linear transition from 40% to 0% A; 8–11 min, 0% A; 11–14 min, linear transition from 0% to 40% A; 14–15 min, linear transition from 40% to 95% A; and 15–18 min, 95% A. The Orbitrap Q Exactive™ mass spectrometer was operated in dual polarity mode (positive and negative) with a spray voltage of 3.2 kV, capillary temperature of 320°C, sheath gas flow rate of 40 arbitrary units, and auxiliary gas flow rate of 10 arbitrary units.

### Raw data processing and metabolite characterization

Raw data files acquired from UHPLC-MS/MS were processed using TraceFinder 3.2.0 (Thermo Fisher) for peak alignment, peak extraction, and metabolite quantification. The processing parameters were configured as follows: retention time tolerance of 0.2 minutes, mass tolerance of 5 ppm, signal-to-noise ratio of 3, and a minimum intensity threshold, among others. Peak intensities were normalized to the total spectral intensity to ensure consistency across samples. Metabolite characterization was achieved by matching the processed peaks against the mzCloud database (https://www.mzcloud.org/) and an in-house reference database, yielding accurate qualitative and relative quantitative results. These metabolites were annotated using the KEGG database (https://www.genome.jp/kegg/pathway.html), HMDB database (https://hmdb.ca/metabolites) and LIPIDMaps database (http://www.lipidmaps.org/). Then the metabolite data was processed by log10 transformation and Pareto scaling.

### Bioinformatics analyses

To classify the four groups, we employed orthogonal partial least squares discriminant analysis (OPLS-DA) using MetaboAnalyst 4.0 (https://www.metaboanalyst.ca/MetaboAnalyst/faces/home.xhtml). This supervised multivariate method maximized the separation between groups and identified key variables contributing significantly to the classification based on their variable importance in projection (VIP) values. Additionally, we used hierarchical clustering to generate dendrograms for patient clustering based on metabolic data, also performed using MetaboAnalyst 4.0. Metabolites with VIP values greater than 1 and false discovery rate (FDR) less than 0.05 were selected as differential metabolites. To identify differential metabolic pathways among the groups, we conducted Kyoto Encyclopedia of Genes and Genomes (KEGG) pathway enrichment analysis on the significantly differential metabolites using MetaboAnalyst 4.0. Heatmaps were generated using the pheatmap package, while bar plots and dot plots were visualized using the ggplot2 package in R.

### Statistical analysis

Patient characteristics were presented by mean ± standard deviation (SD) for continuous variables and by frequency and percentages for categorical variables. Comparisons between ratios were performed using the Chi-square test or Fisher exact test. Continuous variables were analyzed by t-test or nonparametric tests. Receiver operating characteristic (ROC) curve was used to evaluate the diagnostic performance of the variable. P < 0.05 was considered statistically significant. Analysis was performed using statistical package for social science (SPSS) software, version 25.0 (IBM, Armonk, New York, USA).

## Results

### Metabolic profiling of peripheral blood plasma from 4 groups of patients

We followed pregnancy outcome of each patient and categorized all the 94 patients into four groups based on whether they achieved a clinical pregnancy or not. Specifically, the four groups of patients were 16 adenomyosis patients with successful pregnancy (here referred to as the ADM-Success group), 35 adenomyosis patients with failed pregnancy (referred to as the ADM-Fail group), 18 control patients with successful pregnancy (CTRL-Success group), 25 control patients with failed pregnancy (CTRL-Fail group). Clinical pregnancy was determined by the visualization of at least one intrauterine gestational sac using transvaginal sonography 30–35 days after embryo transfer. The demographic and clinical characteristics of all participants are provided in **Supplementary Table S1**.

**Figure 1:**
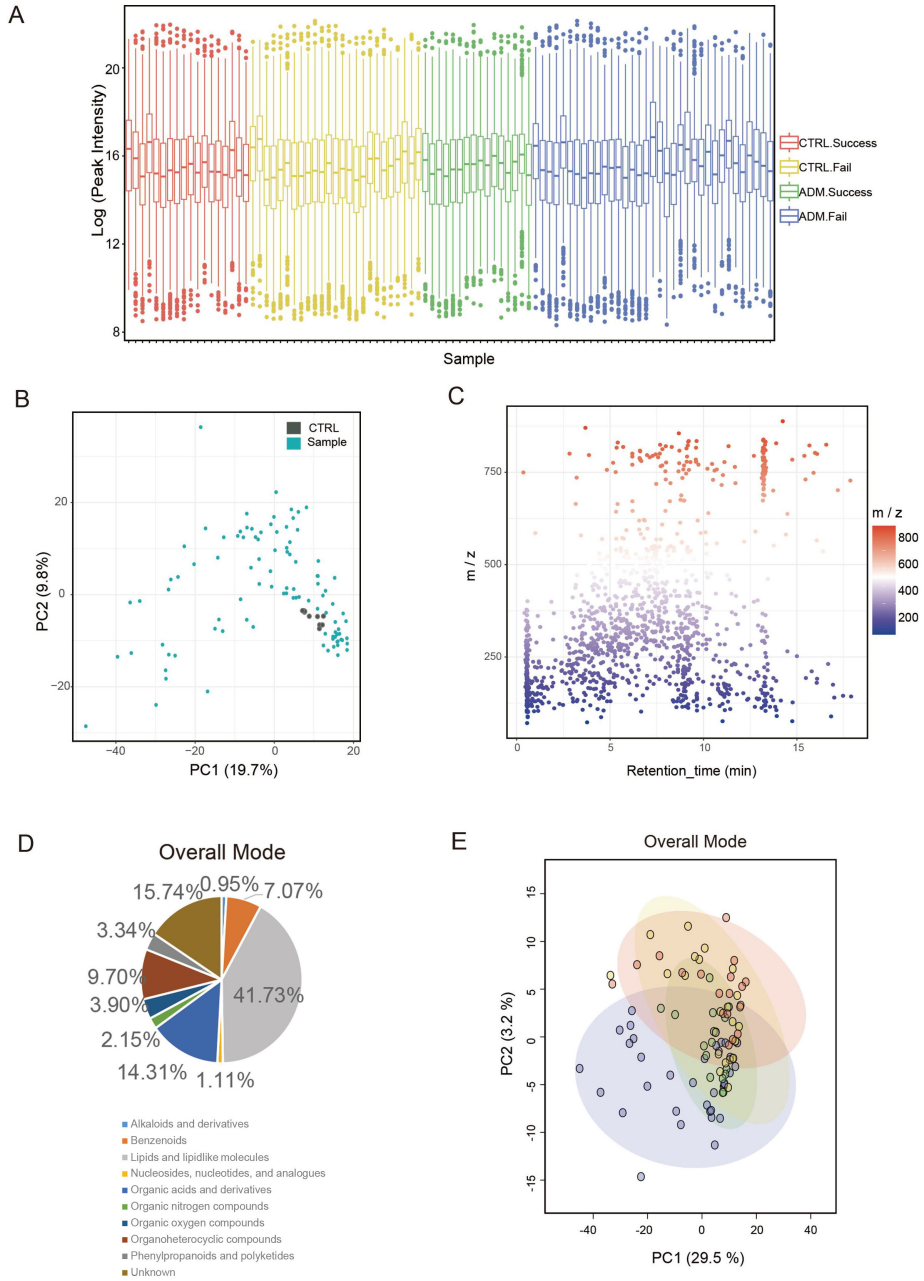
Metabolic profiling of peripheral blood plasma from 4 groups of patients. **(A)** A box plot illustrates the spread of ion signal intensities, with each box corresponding to the metabolite signals from an individual sample. The abundance of ion signals has been transformed using a logarithmic scale (Log e). The box encompasses the interquartile range of metabolite signals, stretching from the first quartile to the third quartile. The median intensity of the metabolites is denoted by the line within the box. Data points lying outside the whiskers are identified as outliers and are represented as separate dots. This box plot reveals no substantial variations in the distribution of ion signals across samples from different groups. **(B)** A principal component analysis (PCA) of the metabolomics dataset is depicted. The green dots represent the samples that were analyzed, while the grey dots indicate the quality control samples. The horizontal and vertical axes correspond to the first and second principal components, respectively, which account for the most significant portions of the data variability. **(C)** Scatter plots illustrate the mass spectral features, defined by their unique mass-to-charge ratios (m/z) and retention times. A total of 1,258 distinct mass spectral features were identified. **(D)** Classes of metabolites detected in overall mode. **(E)** PCA plots of metabolomics data from 4 groups of patients. Each colored dot indicates an individual patient.

After obtaining untargeted metabolomics data, pooling all tested samples and aliquoting them for quality control, boxplots indicate no significant differences between samples from different groups in terms of ion signal distribution (**Figure 1A**). Principal component analysis (PCA) plots with tightly clustered quality control points indicated that the datasets were high quality and the system was reliable (**Figure 1B**).

A total of 1258 mass spectral features (e.g., ions with a specific m/z and retention time) were detected using Reversed Phase Liquid Chromatography (RPLC) electrospray ionization (ESI), i.e. 1258 kinds of recognized compounds were detected (**Figure 1C**). Specifically, there were 784 mass spectral features detected using RPLC ESI positive mode, and 474 features detected using RPLC ESI negative mode, which respectively matched with 784 and 474 recognized compounds in metabolomics databases. Among the compounds identified, top 5 compounds were Lipids and lipidlike molecules, Organic acids and derivatives, Organoheterocyclic compounds, Benzenoids, Organic oxygen compounds (**Figure 1D**). Top 2 compounds in both RPLC ESI positive mode and RPLC ESI negative mode were Lipids and lipidlike molecules and Organic acids and derivatives (**Supplementary Figure S1A**, **Supplementary Figure S1B**).

By PCA plots, distribution pattern of four groups (ADM-Success, ADM-Fail, CTRL-Success, CTRL-Fail) was displayed (**Figure 1E**). In addition, distribution pattern of the four groups in both RPLC ESI positive mode and RPLC ESI negative mode were showed in the figure (**Supplementary Figure S1C**, **Supplementary Figure S1D**). Notably, adenomyosis patients could be distinguished from control group, and pregnant women could be distinguished from un-pregnant women either in adenomyosis group or control group. The majority of ADM-Success samples and CTRL-Success samples formed distinct clusters according to hierarchical clustering, and the majority of ADM-Success samples could be distinguished from ADM-Fail samples (**Supplementary Figure S2**). The above results suggested that four groups of patients had different metabolic profiles.

### Differences in metabolic profiles between adenomyosis and control patients

**Figure 2:**
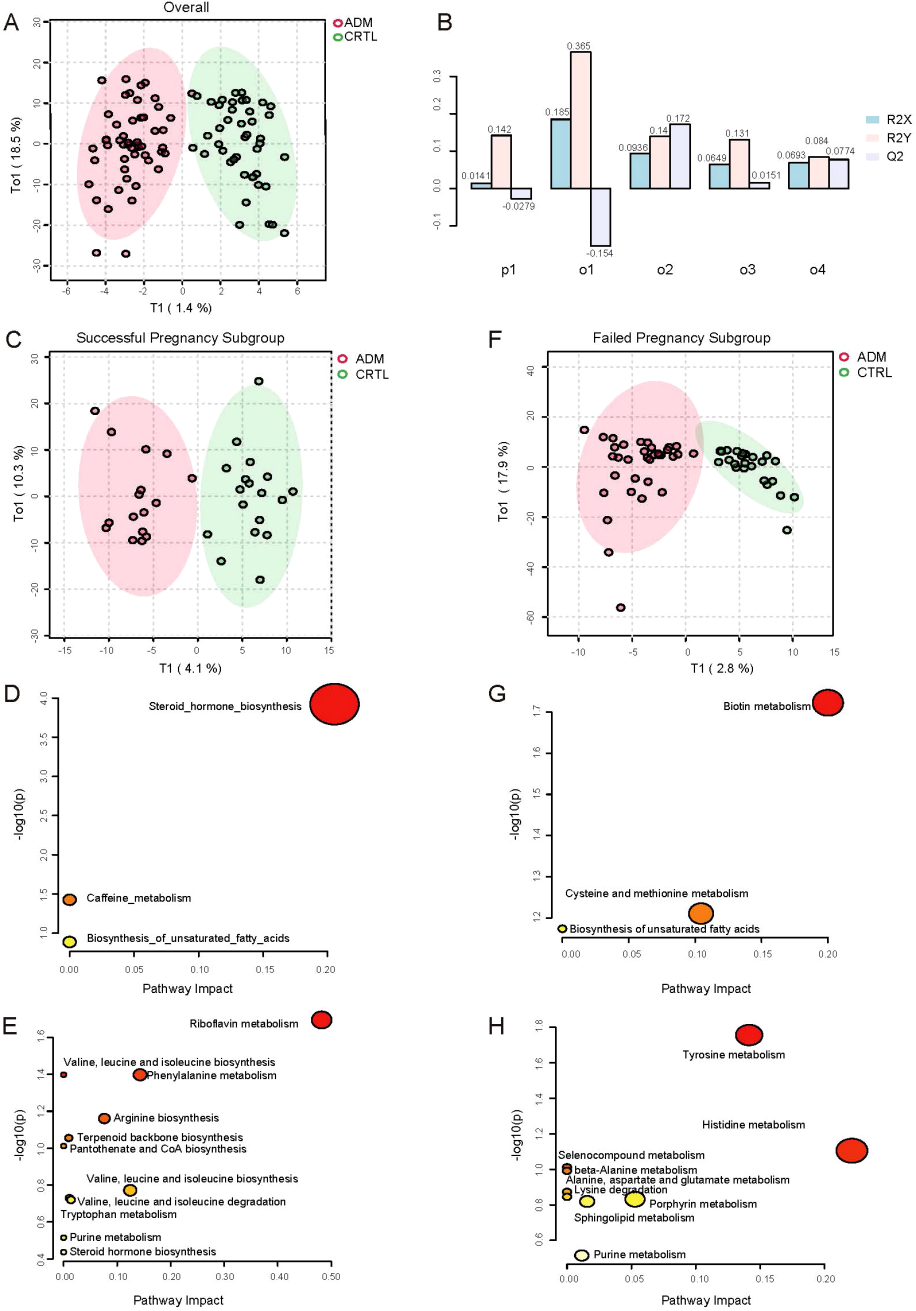
Differences in metabolic profiles between adenomyosis and control patients. **(A)** Orthogonal PLS-DA (OPLS-DA) of metabolomics between adenomyosis and control patients. **(B)** Overview of OPLS Scores for the Normalized Model. The x-axis lists the principal components (p1, o1, o2, o3, o4), each representing a different variable or observation group within the model. The y-axis shows the OPLS scores, which are normalized values ranging from -0.1 to 0.3, indicating the contribution of each component to the model’s predictive ability. The bars are color-coded to represent different conditions or groups: R2X (light blue), R2Y (pink), and Q2 (light purple). The R2X and R2Y scores reflect the explained variation in the X-block (independent variables) and Y-block (dependent variables), respectively, while the Q2 score represents the predicted variation. **(C)** OPLS-DA of metabolomics between adenomyosis and control patients in successful pregnancy subgroup. **(D)** Pathway analysis of differential metabolites between ADM-Success and CTRL-Success group. The up regulated pathways enriched are shown on the y axis, and the x axis represents -log10 (p value). Dot size indicates the impact value of each pathway. **(E)** Pathway analysis of differential metabolites between ADM-Success and CTRL-Success group. The down regulated pathways enriched are shown. **(F)** OPLS-DA of metabolomics between adenomyosis and control patients in failed pregnancy subgroup. **(G)** Pathway analysis of differential metabolites between ADM- Fail and CTRL- Fail group. The up regulated pathways enriched are shown. **(H)** Pathway analysis of differential metabolites between ADM- Fail and CTRL- Fail group. The down regulated pathways enriched are shown.

Firstly, we overall analyzed the metabolic profile characteristics of adenomyosis patients and control patients, irrespective of whether the patients obtained a clinical pregnancy or not. Under orthogonal PLS-DA (OPLS-DA), there appeared to be discrimination between the two groups (**Figure 2A**). The OPLS-DA model’s predictability and fitness were validated through permutation (**Figure 2B**) (**Supplementary Figure S3A**). Our results showed that the difference between the adenomyosis group and the control group was not very significant. A total of 24 metabolites were identified as differential metabolites between two groups, which were with variable important projections (VIPs) >1 and P value <0.05 (**Supplementary Figure S3B**). Among which, 8 kinds of metabolites had increased levels, such as FAHFA, benzoxazepin, LPI, L−glycero−D−manno−Heptose. And the other 16 kinds of metabolites had lower levels in adenomyosis samples compared with control groups, such as PC, Ecgonine methyl ester, SM, Ouabain, Ethyl sorbate and so on (**Supplementary Figure S3B**).

Secondly, given that the difference between the adenomyosis and control groups was not significant in the overall analysis, we did further subgroup analyses, stratifying patients according to whether or not they had achieved a clinical pregnancy. There appeared to be discrimination between the adenomyosis and control patients no matter in successful pregnancy subgroup or failed pregnancy subgroup under OPLS-DA (**Figure 2C**, **Figure 2F**). Validation of the model’s predictability and fitness was performed through permutations (**Supplementary Figure S3C**, **Supplementary Figure S3E**).

In successful pregnancy subgroup, total of 72 metabolites were identified as differential metabolites between adenomyosis and control patients (**Supplementary Figure S3D**). Of those, 24 kinds of metabolites were up-regulated, such as Deoxycorticosterone, Corticosterone, Estradiol, Androsterone and so on. And 48 kinds of metabolites were down-regulated in adenomyosis samples, such as Riboflavin, 3-Methyl-2-oxobutanoic acid, Phenylacetaldehyde and so on (**Supplementary Figure S3D**). Then, we respectively investigated the differential metabolic pathways of up-regulated metabolites and down-regulated metabolites between adenomyosis and control groups through Kyoto Encyclopedia of Genes and Genomes (KEGG) pathway analysis. Up-regulated metabolic pathways included steroid hormone biosynthesis, caffeine metabolism, biosynthesis of unsaturated fatty acids, and the most significant changes were seen in steroid hormone biosynthesis (**Figure 2D**). The top 4 most significantly down-regulated metabolic pathways were riboflavin metabolism, phenylalanine metabolism, arginine biosynthesis, valine, leucine and isoleucine biosynthesis (**Figure 2E**).

In failed pregnancy subgroup, total of 50 metabolites were identified as differential metabolites between two groups (**Supplementary Figure S3F**). In which 21 kinds of metabolites had increased levels, such as Biotin, methionine. The other 29 kinds of metabolites had lower levels in adenomyosis samples compared with control groups, such as Epinephrine, Dopamine, L-Histidine, L-Alanine (**Supplementary Figure S3F**). Similarly, we respectively investigated the up-regulated and down-regulated metabolic pathways between adenomyosis and control groups. Up-regulated metabolic pathways included biotin metabolism, cysteine and methionine metabolism, biosynthesis of unsaturated fatty acids, and biotin metabolism showed to be the most altered metabolic pathways (**Figure 2G**). The top 3 most significantly down-regulated metabolic pathways were tyrosine metabolism, histidine metabolism, porphyrin metabolism (**Figure 2H**).

### Differences in metabolic profiles of adenomyosis patients with successful pregnancy and failed pregnancy

**Figure 3:**
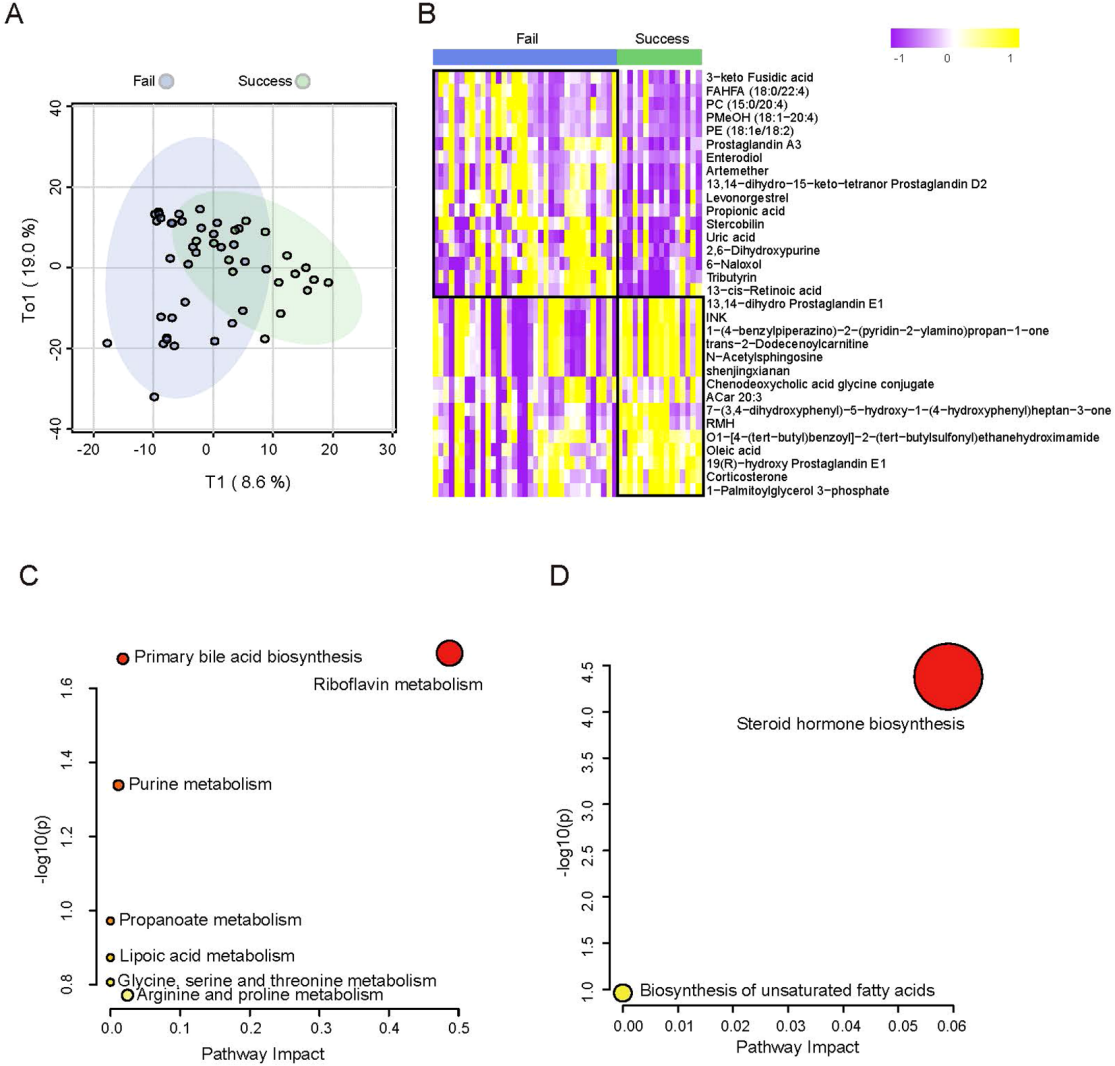
Differences in metabolic profiles of adenomyosis patients with successful pregnancy and failed pregnancy. **(A)** OPLS-DA of metabolomics between adenomyosis patients with successful pregnancy and failed pregnancy. **(B)** Heatmap of plasma metabolomics between patients with ADM-Success and ADM- Fail group. The color schema from violet to yellow represents the relative abundance of metabolites from low to high, respectively. **(C)** Pathway analysis of differential metabolites between ADM-Success and ADM- Fail group. The down regulated pathways enriched are shown. **(D)** Pathway analysis of differential metabolites between ADM-Success and ADM- Fail group. The up regulated pathways enriched are shown.

In this study, we mainly focused on the differences in metabolic profiles between patients who achieved a clinical pregnancy after embryo transfer and those who did not in the adenomyosis cohort. OPLS-DA showed metabolic differences between ADM-Success group and ADM-Fail group (**Figure 3A**). There were 118 kinds of different metabolites between successful pregnancy group and failed pregnancy group in the adenomyosis cohort. When compared with ADM-Fail group, there were 67 metabolites down-regulated and 51 metabolites up-regulated in ADM-Success group. The top-ranked differential metabolites (selected by statistical significance (pvalue<0.05) and abs(log2FoldChange) > 1) were shown in the figure (**Figure 3B**). Different metabolic pathways were then analyzed. The most significantly down-regulated metabolic pathway was Riboflavin metabolism (**Figure 3C**), and the most significantly up-regulated metabolic pathway was Steroid hormone biosynthesis (**Figure 3D**).

### Exploration of predictive model for pregnancy outcome after FET in adenomyosis patients

**Figure 4:**
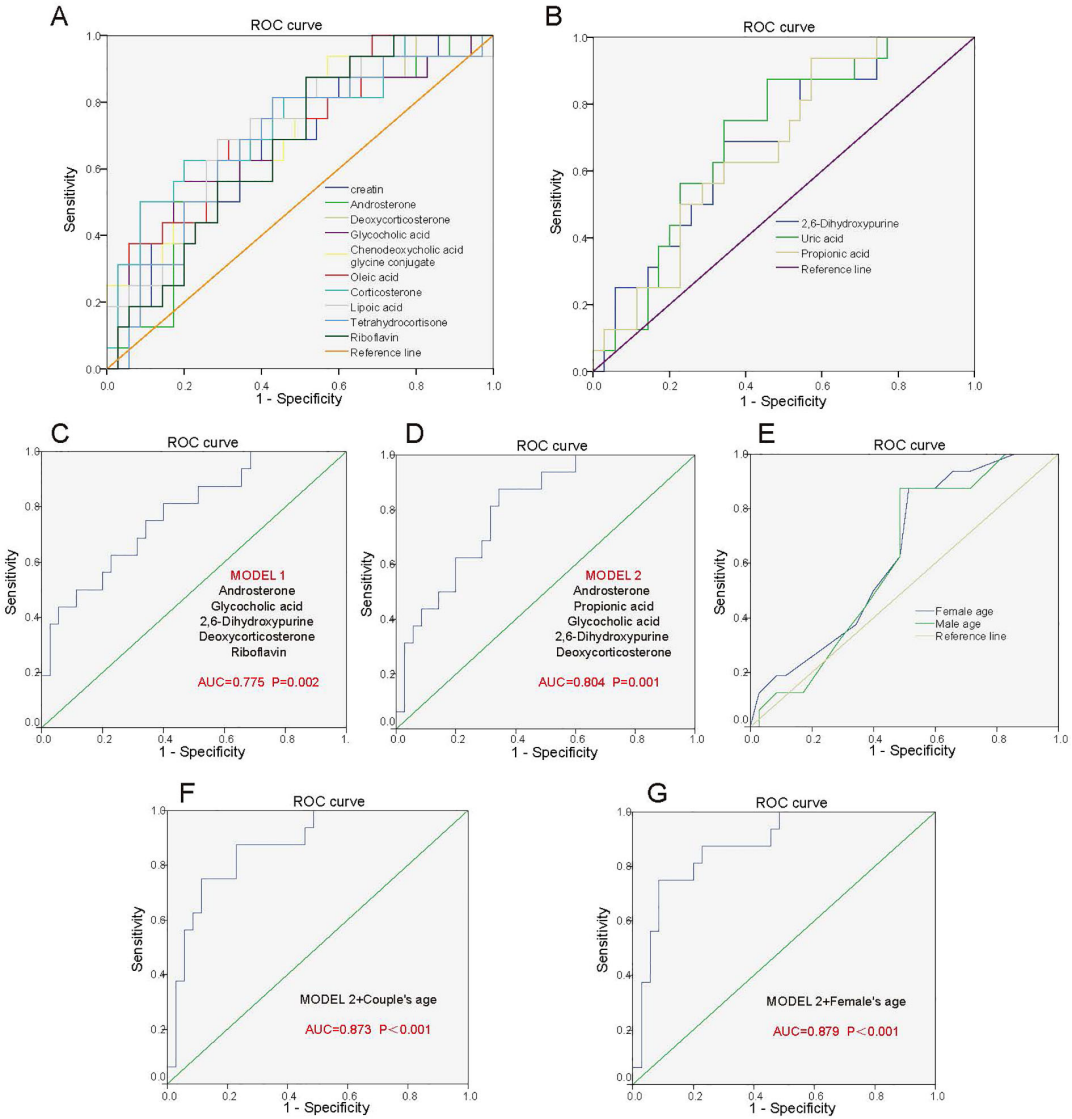
Predictive model for pregnancy outcome after FET in adenomyosis patients. **(A)** ROC analysis of the 10 metabolites for differentiating ADM-Success patients from ADM-Fail patients. 10 metabolites include Tetrahydrocortisone, Riboflavin, Glycocholic acid, Chenodeoxycholic acid glycine conjugate, creatin, Corticosterone, Androsterone, Deoxycorticosterone, Lipoic acid, Oleic acid, which were positively associated with pregnancy outcome of adenomyosis patients. **(B)** ROC analysis of 3 metabolites for differentiating ADM-Success patients from ADM-Fail patients. 3 metabolites include 2,6-Dihydroxypurine, Uric acid, Propionic acid, which were negatively associated with pregnancy outcome of adenomyosis patients. **(C)** Predictive model 1: ROC analysis of 5 metabolites (Androsterone, Glycocholic acid, 2,6-Dihydroxypurine, Deoxycorticosterone, Riboflavin) for differentiating ADM-Success patients from ADM-Fail patients. **(D)** Predictive model 2: ROC analysis of 5 metabolites (Androsterone, Propionic acid, Glycocholic acid, 2,6-Dihydroxypurine, Deoxycorticosterone) for differentiating ADM-Success patients from ADM-Fail patients. **(E)** ROC analysis of the age of the male partner and female partner for differentiating ADM-Success patients from ADM-Fail patients. **(F)** ROC analysis of five metabolites combined with couple’s age for differentiating ADM-Success patients from ADM-Fail patients. **(G)** ROC analysis of five metabolites combined with woman’s age for differentiating ADM-Success patients from ADM-Fail patients.

We intend to establish an effective model for predicting pregnancy outcomes of adenomyosis patients, which can predict pregnancy outcomes 14 days after embryo transfer based on metabolic features in peripheral blood at embryo transfer day. We identified the corresponding metabolites from the enriched metabolic pathways and finally found 13 differential metabolites. The individual diagnostic performances of these metabolites were evaluated by ROC curves. Of these, the levels of 10 metabolites (Tetrahydrocortisone, Riboflavin, Glycocholic acid, Chenodeoxycholic acid glycine conjugate, Creatin, Corticosterone, Androsterone, Deoxycorticosterone, Lipoic acid, Oleic acid) were positively associated with pregnancy outcome of adenomyosis patients, and the levels of 3 metabolites (2,6-Dihydroxypurine, Uric acid, Propionic acid) were negatively associated. The above metabolites individually yielded areas under the curve (AUCs) of 0.673-0.738 (**Figure 4A**, **Figure 4B**). Further, we screened for metabolites with high diagnostic performances. Two screening strategies were used to construct predictive models of pregnancy outcomes. Firstly, we selected metabolic pathways that were significantly different between the two groups, and one kind of important metabolite in each metabolic pathway. Because the steroid hormone biosynthesis pathway was the most significant different pathway between the groups, we selected 2 important metabolites in the steroid hormone biosynthesis pathway. Then, a total of 5 metabolites (Androsterone, Glycocholic acid, 2,6-Dihydroxypurine, Deoxycorticosterone, Riboflavin) were included in the prediction model. The AUC for the combination of the above five metabolites to predict pregnancy outcome in adenomyosis was 0.775 (P=0.002) (**Figure 4C**). Secondly, we selected the TOP 5 metabolites (Androsterone, Propionic acid, Glycocholic acid, 2,6-Dihydroxypurine, Deoxycorticosterone), which were with the most significant differences between groups, and all of these metabolites were with important biological functions in the corresponding pathway. The AUC for the combination of the above five metabolites was 0.804 (P=0.001), which had an obviously higher diagnostic value in identifying patients with successful pregnancy from patients with failed pregnancy (**Figure 4D**).

Age of the couple is strongly associated with pregnancy outcome, especially the age of the female partner, and we analyzed the diagnostic performances of the age of the male partner and female partner on pregnancy outcome by ROC. The AUC for the age of the female partner and male partner were 0.637 and 0.613, respectively (**Figure 4E**). Subsequently, we selected five metabolites with most significant differences between groups and combined age with the metabolites to predict pregnancy outcomes. The AUC of the five differential metabolites combined with couple’s age was 0.873 (P<0.001) (**Figure 4F**). The AUC of the five differential metabolites combined with woman’s age was 0.879 (P<0.001), which was better than combined with couple’s age (**Figure 4G**). Performance evaluation metrics (cut-off values, AUC, sensitivity and specificity) for the above different prediction models were listed in the **Supplementary Table S2**. In a word, the “5 metabolites + age (i.e. 5 metabolites combined with woman’s age)” panel could effectively predict pregnancy outcomes.

## Discussion

This study compared the metabolic differences between ADM-Success group and ADM-Fail group, and established a “5 metabolites + age” panel, which could effectively predict pregnancy outcomes of adenomyosis patients. And this study explored the metabolic differences between adenomyosis group and control group.

Main metabolic pathways that differed significantly between the two groups were Steroid hormone biosynthesis, Riboflavin metabolism, Biotin metabolism, Tyrosine metabolism. The major metabolites corresponding to the above pathways were Deoxycorticosterone, Corticosterone, Androsterone, Estradiol, Riboflavin, Biotin and Tyrosine. These metabolites are closely related to inflammation and immunity (11–16). Previous metabolism-related studies have also identified an important role for steroid hormone biosynthesis in the pathogenesis of adenomyosis (17, 18). Peipei Chen et al. found sixty differential expressed metabolites in intestinal metabolites from adenomyosis-female ICR mice and control groups, which were mainly involved in steroid hormone biosynthesis (5). Deoxycorticosterone, a precursor of aldosterone, is one of the mineralocorticoid produced by the adrenal cortex. Yan Deng et al. showed that an increase in deoxycorticosterone can lead to pathological cardiac hypertrophy, hypertension and increased inflammation in mice (19). Corticosterone, one of the glucocorticoids produced by the adrenal cortex, was shown to increase levels of pro-inflammatory cytokines in mouse microglia and was associated with depressive behavior in mice (20). Similarly, Patrizia Carrarelli et al. also found that endometrium of adenomyosis patients showed high expression of corticotropin-releasing hormone when compared with control patients, which was consistent with the increased Deoxycorticosterone and Corticosterone (21). Androsterone is a precursor substance of testosterone and could furtherly convert to estradiol. Estradiol could upregulate COX-2 levels and promote prostaglandin E2 (PGE2) production, which were strongly associated with the development of adenomyosis. Riboflavin, also known as vitamin B2, is a water-soluble vitamin, with antioxidant and anti-inflammatory properties and could improve inflammatory bowel disease (22). Biotin, also known as vitamin B7, is involved in immune and inflammatory regulation, including fatty acid biosynthesis, regulation of lymphocyte and macrophage gene expression, regulation of T-cell maturation and function, biotinylation of proteins, and chemokine production (23). Tyrosine could also exert anti-inflammatory effects, and its metabolite p-cresol sulfate can reduce allergic airway inflammation by selectively inhibiting CCL20 production by airway epithelial cells (24). Overall, the above hormones, vitamins and amino acids have pro-inflammatory/anti-inflammatory effects, and the development of adenomyosis is closely related to estrogen and is a chronic inflammatory disease, thus it is hypothesized that the above metabolites may also lead to the infiltration of immune cells and the release of cytokines, which may promote the growth and invasion of endometrial glands into the myometrium, however the detailed molecular mechanisms need to be confirmed by further basic medical research.

**Figure 5:**
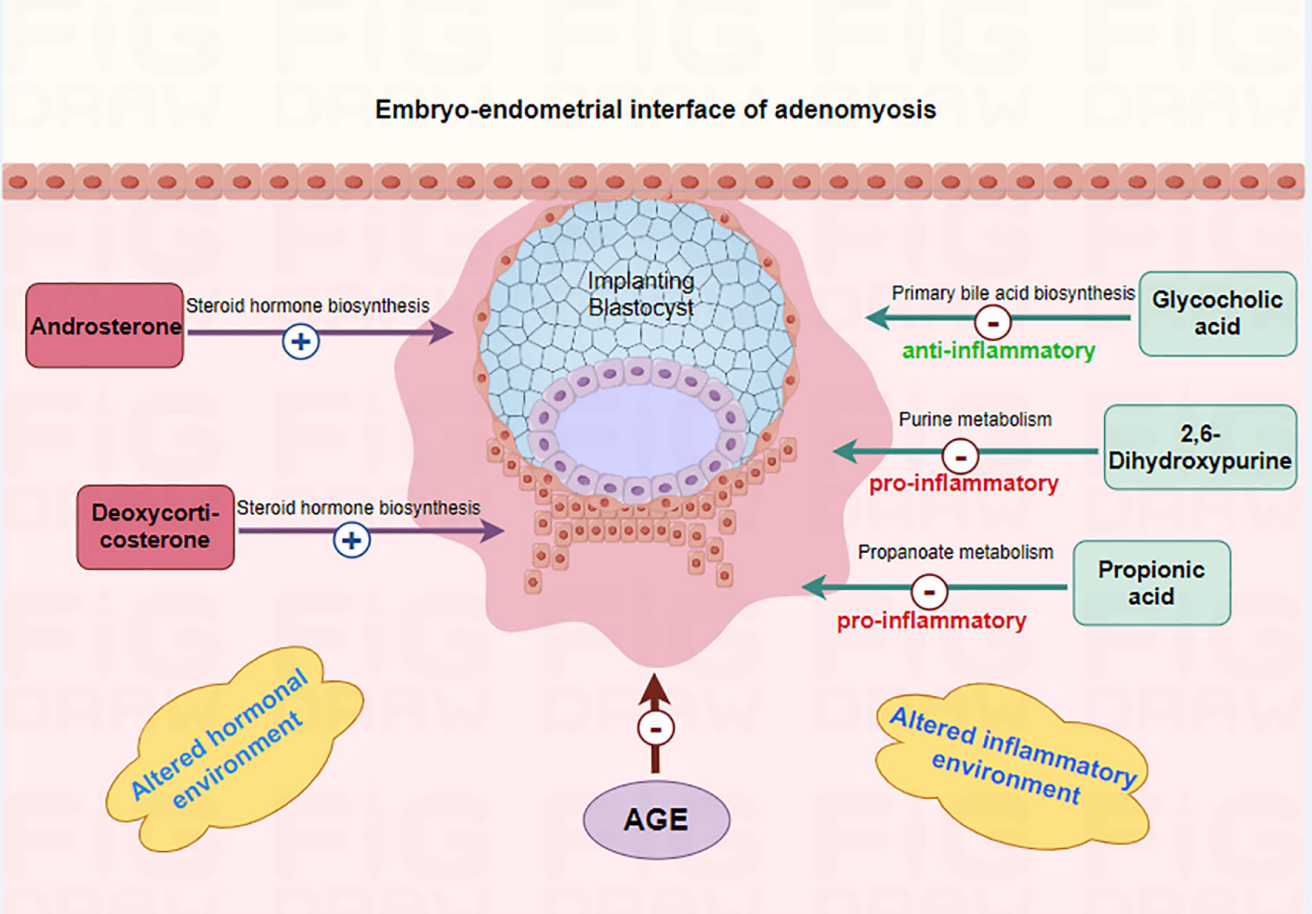
Mechanism of differential metabolites affecting embryo implantation in adenomyosis-associated infertile patients. The 5 metabolites (Androsterone, Propionic acid, Glycocholic acid, 2,6-Dihydroxypurine, Deoxycorticosterone) may affect endometrial receptivity and embryo implantation by altering the inflammatory and hormonal environments of the endometrium.

This study found that “5 metabolites + age” panel could effectively predict pregnancy outcomes of adenomyosis patients. The 5 metabolites included Androsterone, Propionic acid, Glycocholic acid, 2,6-Dihydroxypurine, Deoxycorticosterone. Androsterone, Deoxycorticosterone levels were higher and Propionic acid, Glycocholic acid, 2,6-Dihydroxypurine levels were lower in ADM-Success group as compared to ADM-Fail group. The above five metabolites can affect pregnancy outcomes of adenomyosis patients through sex hormone regulation, inflammatory regulation and other pathways (**Figure 5**). The function of Androsterone, Deoxycorticosterone is as previously described. Propionic acid is a short-chain fatty acid with anti-inflammatory and immune homeostatic regulatory properties. Hongzhen Chen et al. showed that compared with the PBS-treated group, the transcription level of inflammation-related genes (MYD88, NF-κB, ICAM, PTPN2, and TLR3), chemokines and inflammatory cytokines (CXCL11, TNFα, CCL2, IL-6, and CXCL8), as well as migration- and invasion-related genes, were down-regulated in rheumatoid arthritis -fibroblast-like synoviocytes treated with propionate (25). Glycocholic acid is a conjugated bile acid formed by the combination of bile acids and glycine, which is a type of bile acid. Elevated bile acids stimulate the uterus, releasing prostaglandins and inducing uterine contractions, which may be the reason why higher levels of glycocholic acid had been associated with pregnancy failure after embryo transfer in adenomyosis patients. 2,6-Dihydroxypurine can be generated from hypoxanthine catalyzed by xanthine oxidase (XO). In this process, XO can generate reactive oxygen species (ROS) such as superoxide anion (O2-) and H2O2. ROS are powerful oxidizing agents that can lead to cell and tissue damage and have been shown to induce the release of multiple inflammatory mediators, such as cytokines and chemokines, which exacerbate the inflammatory response (26). Overall, the above metabolic molecules may affect endometrial receptivity and embryo implantation by altering the inflammatory and hormonal environments of the endometrium. The effect of the above metabolites is summarized in the **Figure 5**. However, the specific molecular mechanisms need to be further confirmed in subsequent studies. Nevertheless, the predictive value of the 5 metabolites on pregnancy outcome is clear. In this study, the AUC of the 5 metabolites combined with age was 0.879 and the P value was <0.001, which has a good clinical application value.

In a word, we innovatively explored the metabolic differences in peripheral blood on the embryo transfer day between adenomyosis and controls, as well as differences in metabolic profiles between ADM-Success group and ADM-Fail group. What is more, a prediction model of plasma metabolic profiles for predicting pregnancy outcome in adenomyosis patients was constructed, which was a minimally invasive predictor and was meaningful for clinical practice. However there are some limitations in this study. Firstly, a validation cohort needs to be constructed to verify the reliability of the above findings. Secondly, basic medical experimental studies should be conducted to verify the action mechanism of the relevant metabolites, especially for the metabolites that are strongly associated with pregnancy outcomes in adenomyosis.

## Conclusion

A “5 metabolites + age” panel could effectively predict pregnancy outcomes of adenomyosis patients who undergoing FET. There were differences in plasma metabolic profiles between adenomyosis-associated infertility and control patients.

## Data Availability

The raw data supporting the conclusions of this article will be made available by the authors, without undue reservation.

## Supplementary material

Supplementary Figure 1 Metabolic profiling of peripheral blood plasma from 4 groups of patients. **(A, B)** Classes of metabolites detected in RPLC ESI positive mode and RPLC ESI negative mode. **(C, D)** PCA plots of metabolomics data from 4 groups of patients in RPLC ESI positive mode and RPLC ESI negative mode. Each colored dot indicates an individual patient.

Supplementary Figure 2 Metabolic profiling of peripheral blood plasma from 4 groups of patients. **(A)** Dendrogram of hierarchical clustering of metabolomics data from 4 groups of patients in overall mode. **(B)** Dendrogram of hierarchical clustering of metabolomics data from 4 groups of patients in RPLC ESI positive mode. **(C)** Dendrogram of hierarchical clustering of metabolomics data from 4 groups of patients in RPLC ESI negative mode.

Supplementary Figure 3 Differences in metabolic profiles between adenomyosis and control patients. **(A)** The validity of OPLS-DA model (Adenomyosis VS Control) was confirmed by the permutation test, showing no overfitting. **(B)** Heatmap of plasma metabolomics between Adenomyosis and Control patients. **(C)** The validity of OPLS-DA model (ADM-Success group VS CTRL-Success group) was confirmed by the permutation test, showing no overfitting. **(D)** Heatmap of plasma metabolomics between ADM-Success group and CTRL-Success group. **(E)** The validity of OPLS-DA model (ADM-Fail group VS CTRL-Fail group) was confirmed by the permutation test, showing no overfitting. **(F)** Heatmap of plasma metabolomics between ADM-Fail group and CTRL-Fail group.
