# Supplementary figures and images for: Profiling the metabolome of adenomyosis-associated infertility patients to predict the pregnancy outcome of frozen embryo transfer
Source: Front Endocrinol (Lausanne). 2025 Aug 25;16:1625638. doi: 10.3389/fendo.2025.1625638 (PMC12414768; doi:10.3389/fendo.2025.1625638)

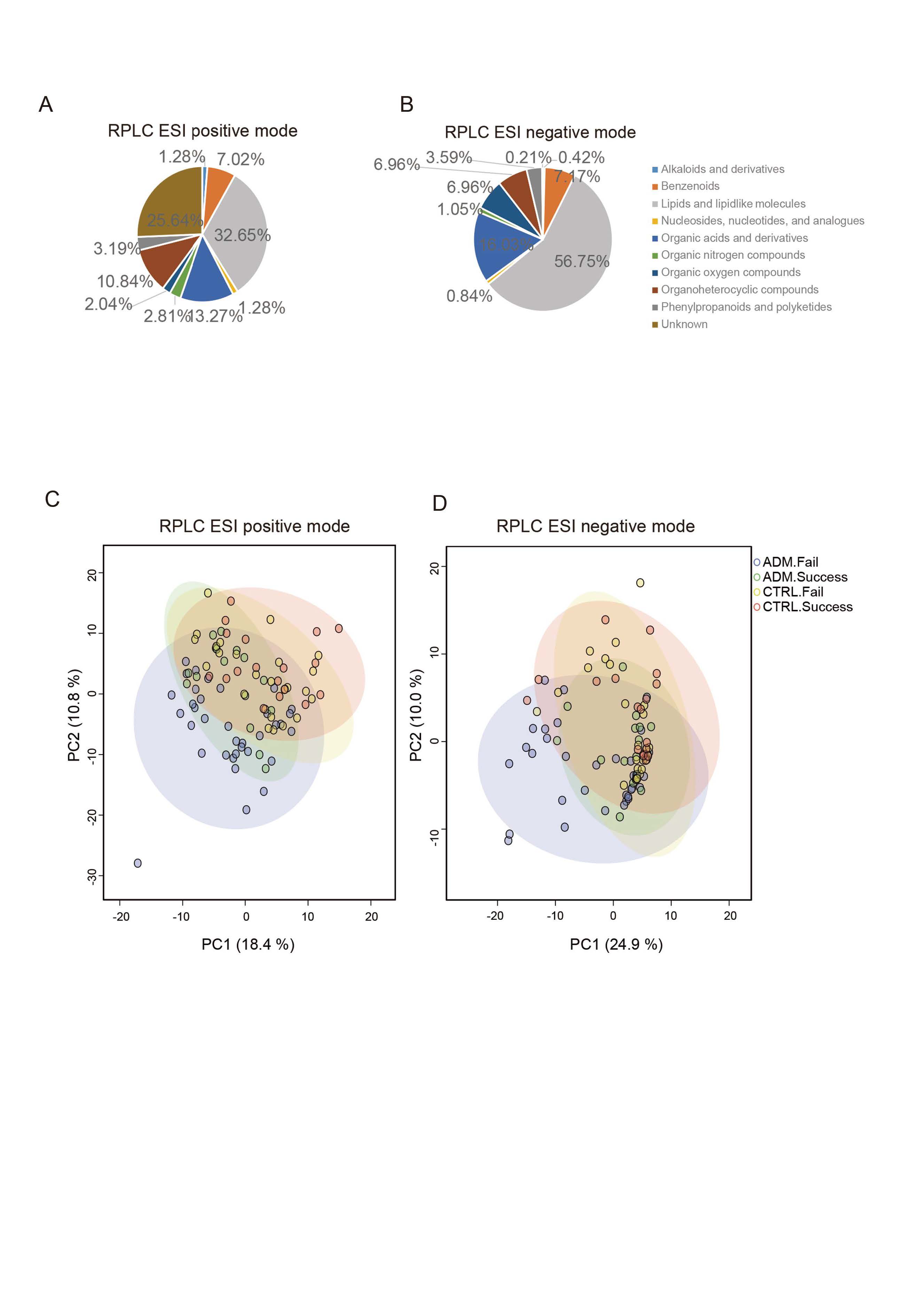

Supplement: Supplementary Figure 1 — Metabolic profiling of peripheral blood plasma from 4 groups of patients. (A, B) Classes of metabolites detected in RPLC ESI positive mode and RPLC ESI negative mode. (C, D) PCA plots of metabolomics data from 4 groups of patients in RPLC ESI positive mode and RPLC ESI negative mode. Each colored dot indicates an individual patient. [file Image1.jpeg]

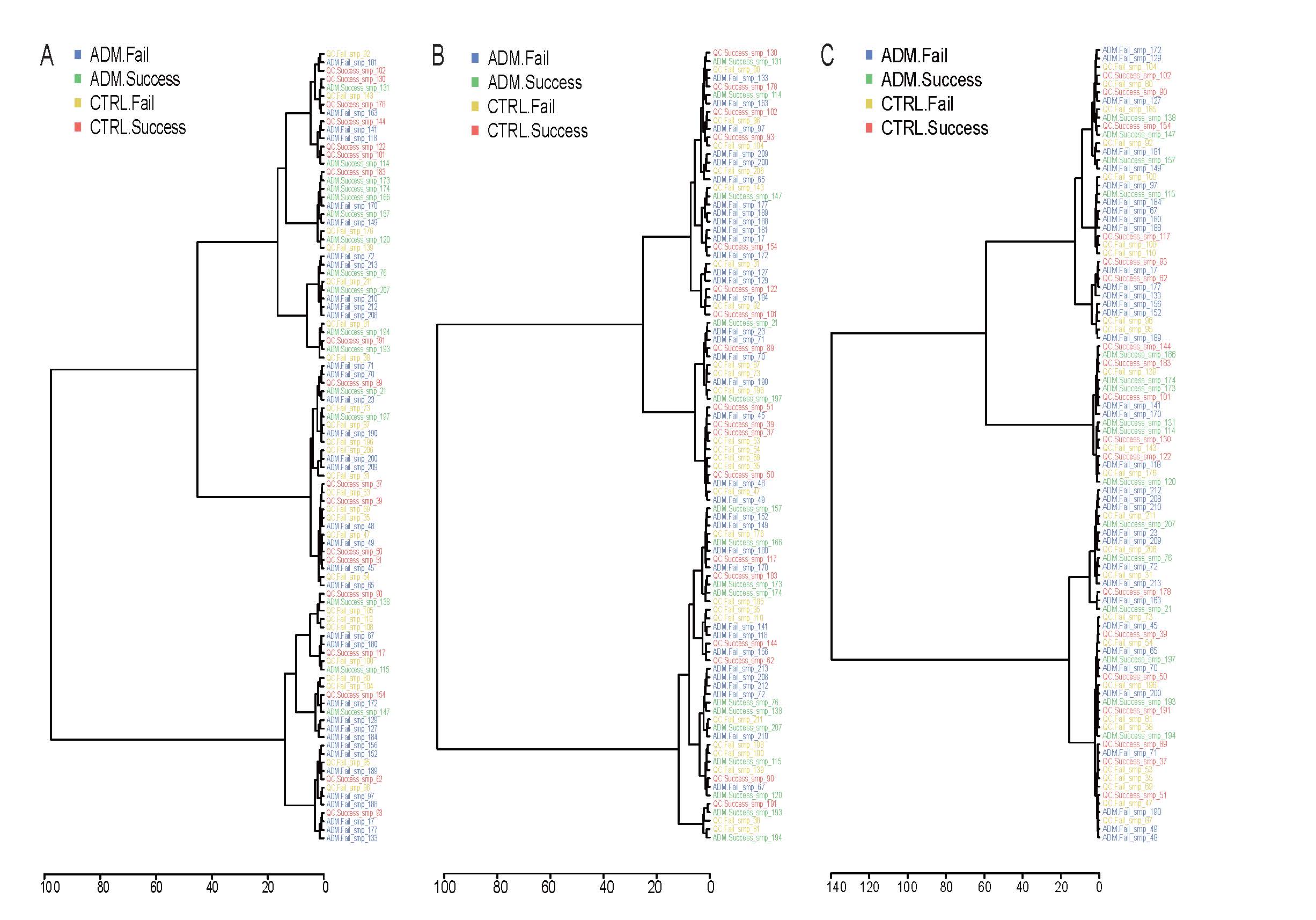

Supplement: Supplementary Figure 2 — Metabolic profiling of peripheral blood plasma from 4 groups of patients. (A) Dendrogram of hierarchical clustering of metabolomics data from 4 groups of patients in overall mode. (B) Dendrogram of hierarchical clustering of metabolomics data from 4 groups of patients in RPLC ESI positive mode. (C) Dendrogram of hierarchical clustering of metabolomics data from 4 groups of patients in RPLC ESI negative mode. [file Image2.jpeg]

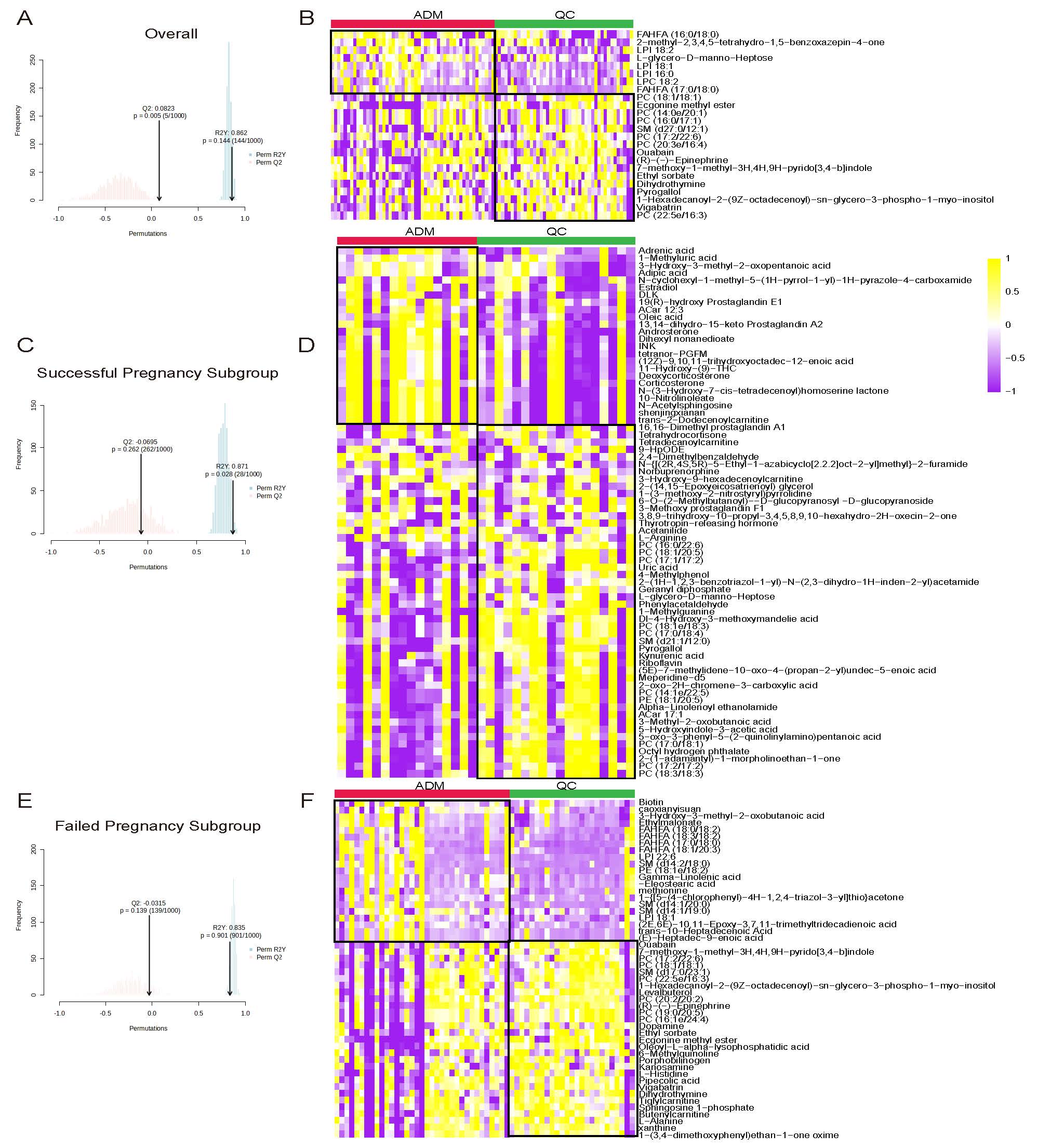

Supplement: Supplementary Figure 3 — Differences in metabolic profiles between adenomyosis and control patients. (A) The validity of OPLS-DA model (Adenomyosis VS Control) was confirmed by the permutation test, showing no overfitting. (B) Heatmap of plasma metabolomics between Adenomyosis and Control patients. (C) The validity of OPLS-DA model (ADM-Success group VS CTRL-Success group) was confirmed by the permutation test, showing no overfitting. (D) Heatmap of plasma metabolomics between ADM-Success group and CTRL-Success group. (E) The validity of OPLS-DA model (ADM-Fail group VS CTRL-Fail group) was confirmed by the permutation test, showing no overfitting. (F) Heatmap of plasma metabolomics between ADM-Fail group and CTRL-Fail group. [file Image3.jpeg]
