# Supplementary material for: Profiling the metabolome of adenomyosis-associated infertility patients to predict the pregnancy outcome of frozen embryo transfer
Source: Front Endocrinol (Lausanne). 2025 Aug 25;16:1625638. doi: 10.3389/fendo.2025.1625638 (PMC12414768; doi:10.3389/fendo.2025.1625638)
Supplement: Supplementary file 4 [file Table1.docx]

Table S1 Demographic and clinical characteristics of included participants

|  | **Adenomyosis group** | | **Control group** | |
| --- | --- | --- | --- | --- |
|  | ADM-Success  N=16 | ADM-Fail  N=35 | CTRL-Success  N=18 | CTRL-Fail  N=25 |
| **Clinical characteristics** | | | | |
| Female age (yr) (mean±SD) | 33.4±2.9 | 35.5±3.9 | 33.4±4.4 | 35.8±4.0 |
| Male age (yr) (mean±SD) | 34.5±2.8 | 36.6±4.4 | 35.4±4.7 | 36.5±4.7 |
| Infertility type (%) |  |  |  |  |
| Primary infertility | 13（13.8%） | 23（24.5%） | 10（10.6%） | 11（11.7%） |
| Secondary infertility | 3（3.2%） | 12（12.8%） | 8（8.5%） | 14（14.9%） |
| Infertility duration (yr)(median (quartile)) | 3 （2.5,4） | 4（5，6.5） | 3（1，4） | 3（2，5） |
| BMI（kg/m^2^）(mean±SD) | 23.0±3.2 | 22.5±2.4 | 21.5±3.7 | 22.7±3.3 |
| AMH（ng/mL）(median (quartile)) | 1.6（1.2，3.0） | 1.6（1.3，2.0） | 2.5（1.2，3.1） | 1.4（0.8，2.0） |
| AFC (median (quartile)) | 10.5（7，13.5） | 11（7，13） | 12（9，19） | 10（5，15） |
|  |  |  |  |  |
| **FET characteristics** | | | | |
| FET protocol (%) |  |  |  |  |
| Artificial cycle | 13（13.8%） | 32（34.0%） | 9（9.6%） | 14（14.9%） |
| Natural cycle | 3（3.2%） | 3（3.2%） | 9（9.6%） | 11（11.7%） |
| Endometrial thickness（mm）(mean±SD) | 9.3±1.7 | 8.3±0.9 | 8.8±0.9 | 8.8±1.0 |
| Embryo/Blastocyst (%) |  |  |  |  |
| Embryo | 6（6.4%） | 15（16.0%） | 6（6.4%） | 7（7.4%） |
| Blastocyst | 10（10.6%） | 20（21.3%） | 12（12.8%） | 18（19.1%） |
| Number of embryo/blastocyst (mean±SD) | 1.3±0.5 | 1.4±0.5 | 1.3±0.5 | 1.3±0.4 |
| **FET pregnancy outcome** | | | | |
| Clinical pregnancy rate（%） | 31.4% | | 41.9% | |

**BMI**: body mass index. **AMH**: Anti-Mullerian Hormone. **AFC**: antral follicle count. **FET**: frozen embryo transfer.
