# Supplementary material for: Profiling the metabolome of adenomyosis-associated infertility patients to predict the pregnancy outcome of frozen embryo transfer
Source: Front Endocrinol (Lausanne). 2025 Aug 25;16:1625638. doi: 10.3389/fendo.2025.1625638 (PMC12414768; doi:10.3389/fendo.2025.1625638)
Supplement: Supplementary file 5 [file Table2.docx]

Table S2 Performance evaluation metrics for different prediction models

| **Indicator** | **Cut - off value** (Peak Area Integration) | **AUC** | **Sensitivity** | **Specificity** |
| --- | --- | --- | --- | --- |
| **Single-indicator Prediction Model** | | | | |
| Creatin | 16282431.1 | 0.682 | 0.500 | 0.829 |
| Androsterone | 9697174.9 | 0.677 | 0.688 | 0.714 |
| Deoxycorticosterone | 9820704.2 | 0.686 | 0.563 | 0.657 |
| Glycocholic acid | 3665735.6 | 0.686 | 0.563 | 0.829 |
| Chenodeoxycholic acid glycine conjugate | 4173703.7 | 0.704 | 0.938 | 0.429 |
| Oleic acid | 12843406.2 | 0.725 | 0.688 | 0.686 |
| Corticosterone | 17656950.8 | 0.738 | 0.625 | 0.800 |
| Lipoic acid | 32970450.4 | 0.707 | 0.688 | 0.714 |
| Tetrahydrocortisone | 1145516.4 | 0.686 | 0.813 | 0.571 |
| Riboflavin | 236126.9 | 0.673 | 0.875 | 0.486 |
| 2,6-Dihydroxypurine | 7598938.6 | 0.684 | 0.688 | 0.657 |
| Uric acid | 34358472.3 | 0.705 | 0.875 | 0.543 |
| Propionic acid | 473496.5 | 0.673 | 0.938 | 0.429 |
| Female age (yr) | 36.0 | 0.637 | 0.875 | 0.486 |
| Male age (yr) | 37.0 | 0.613 | 0.875 | 0.514 |
| **Combined-indicator Prediction Model** | | | | |
| Model 1 | / | 0.775 | 0.813 | 0.600 |
| Model 2 | / | 0.804 | 0.875 | 0.657 |
| MODEL 2+Couple's age | / | 0.873 | 0.875 | 0.771 |
| MODEL 2+Female's age | / | 0.879 | 0.750 | 0.914 |

Peak Area Integration: Represents the response intensity of the metabolite and indicates the relative content of the metabolite.

AUC: Area under the curve.

Model 1: Including 5 metabolites: Androsterone, Glycocholic acid, 2,6-Dihydroxypurine, Deoxycorticosterone, Riboflavin.

Model 2: Including 5 metabolites: Androsterone, Propionic acid, Glycocholic acid, 2,6-Dihydroxypurine, Deoxycorticosterone.
